# Testosterone Therapy in a Patient With Fluoroquinolone-Associated Tendinopathy: A Case Report of Rapid-Onset Achilles Injury Following Ciprofloxacin

**DOI:** 10.7759/cureus.114935

**Published:** 2026-08-21

**Authors:** Khashayar Farzam

**Affiliations:** 1 Metabolic Health, True North Metabolic, Kitchener, CAN; 2 Health Sciences, McMaster University, Kitchener, CAN

**Keywords:** achilles tendinopathy, adverse drug reaction, ciprofloxacin, fluoroquinolone, men's health, sports medicine, tendon injury, testosterone, testosterone replacement therapy (trt)

## Abstract

Testosterone replacement therapy (TRT) is increasingly used in men with testosterone deficiency, and emerging observational data have suggested an association between therapeutic testosterone exposure and tendon injury. Fluoroquinolones are independently associated with tendinopathy and tendon rupture, particularly involving the Achilles tendon. We report a man in his 30s receiving physiologic TRT who developed severe, rapidly progressive bilateral Achilles tendon symptoms shortly after starting ciprofloxacin 500 mg twice daily for a urinary tract infection with suspected prostatic and upper urinary tract involvement. Testosterone therapy had been initiated approximately two months earlier and was continued unchanged throughout the event and recovery. Ciprofloxacin was discontinued after the onset of tendon symptoms, followed by gradual clinical improvement over several weeks. The close temporal relationship with ciprofloxacin exposure and subsequent improvement after withdrawal supports ciprofloxacin as the likely acute precipitating factor. Concurrent physiologic TRT may have represented an additional susceptibility factor; however, a causal interaction between testosterone therapy and fluoroquinolones has not been established. This case highlights an unusually rapid and severe presentation of fluoroquinolone-associated Achilles tendinopathy and supports further investigation into whether therapeutic androgen exposure may modify tendon vulnerability in patients receiving fluoroquinolones.

## Introduction

Testosterone replacement therapy (TRT) is used to restore physiologic androgen concentrations in men with testosterone deficiency. Emerging observational evidence has associated prescription testosterone therapy with injuries involving several tendons, including the Achilles tendon, although these studies do not establish a direct causal effect of physiologic testosterone on tendon injury [1-5]. The biological relationship between androgens and tendon tissue remains incompletely understood. Proposed mechanisms include alterations in collagen turnover, extracellular matrix remodeling, and the adaptation of tendon tissue to mechanical loading. Much of the mechanistic literature involves supraphysiologic anabolic-androgenic steroid exposure and therefore cannot be directly extrapolated to physiologic TRT. Experimental findings have also been inconsistent, with androgen exposure associated with impaired tendon adaptation in some models and potentially favorable effects on tendon healing in others [6-8].

Fluoroquinolone-associated tendon toxicity is more firmly established. Ciprofloxacin and other systemic fluoroquinolones are associated with tendinopathy and tendon rupture, particularly involving the Achilles tendon, and this risk is recognized in regulatory warnings and epidemiologic literature [9-13]. Proposed mechanisms include impaired tenocyte function, disruption of collagen synthesis and cross-linking, altered extracellular matrix turnover, and increased matrix degradation [14,15].

Whether concurrent physiologic testosterone therapy modifies susceptibility to fluoroquinolone-associated tendon injury has received little direct study. Importantly, available evidence does not establish a pharmacologic interaction between testosterone and fluoroquinolones. We report a case of rapidly developing, functionally disabling bilateral Achilles tendinopathy following ciprofloxacin exposure in a man in his 30s receiving physiologic TRT. Ciprofloxacin was considered the likely acute precipitating factor, while concurrent testosterone therapy is presented only as a possible and unproven susceptibility factor.

## Case presentation

A man in his 30s with testosterone deficiency had initiated TRT approximately two months before presentation. He was receiving testosterone cypionate 16 mg daily, equivalent to 112 mg weekly, with follow-up testosterone concentrations maintained within the physiologic range. He had no prior history of Achilles tendinopathy, tendon rupture, or other clinically significant tendon disorder. He had no pertinent medical history, had a body mass index within the healthy range, and was a nonsmoker. He had no history of diabetes mellitus, chronic kidney disease, or rheumatologic disease and was not receiving corticosteroid or statin therapy. He denied use of anabolic-androgenic steroids, other androgenic compounds, or aromatase inhibitors. His only other regular medication was finasteride 1 mg daily for androgenetic alopecia. There had been no recent strenuous exercise, unusual physical activity, or other mechanical loading that would be expected to predispose him to Achilles tendinopathy.

In late June, approximately two months after initiation of testosterone therapy, the patient developed urinary symptoms. Initial urine testing demonstrated pyuria. Nitrofurantoin was prescribed initially, but because of an inadequate clinical response and concern for prostatic or upper urinary tract involvement, antimicrobial therapy was changed to ciprofloxacin 500 mg twice daily.

No significant musculoskeletal symptoms occurred after the first dose of ciprofloxacin. After the second dose, however, the patient developed new pain involving the knees, ankles, and several additional musculoskeletal sites. He took a third dose the following morning. Within several hours, he developed severe pain localized predominantly to the right Achilles tendon, followed by substantial involvement of the contralateral Achilles tendon. The symptoms progressed rapidly and resulted in marked difficulty with ambulation. The patient reported significant pain with weight-bearing and worsening Achilles pain with movement of the feet, making walking difficult. He was unable to drive and required wheelchair assistance for transportation. He also reported milder discomfort involving tendons of the upper extremities, although the predominant symptoms and functional limitation involved the Achilles tendons.

There was no evidence of Achilles tendon rupture, and there was also evidence of diffuse tendon involvement rather than isolated symptoms to the Achilles tendons. The presentation was considered clinically consistent with acute Achilles tendinopathy. As tendinopathy is primarily a clinical diagnosis and there was no evidence suggesting rupture or another complication requiring urgent structural assessment, diagnostic imaging was not considered immediately necessary. Renal function and liver enzymes were normal, and the patient did not have any history of hyperglycemia.

Given the close temporal relationship between ciprofloxacin exposure and the onset of severe bilateral Achilles symptoms, fluoroquinolone-associated tendinopathy was suspected, and ciprofloxacin was discontinued. TRT was continued without interruption at the same dose.

Following ciprofloxacin withdrawal, Achilles tendon pain and functional impairment gradually improved. Over the course of several weeks, the patient progressively regained function despite continued testosterone therapy. There was no subsequent evidence of Achilles tendon rupture and no recurrence of a similar acute tendon syndrome while TRT was continued.

## Discussion

This case describes rapid-onset bilateral Achilles tendinopathy shortly after ciprofloxacin exposure in a man in his 30s receiving physiologic TRT. The temporal sequence strongly implicates ciprofloxacin as the acute precipitating exposure. The patient had tolerated TRT for approximately two months without tendon symptoms, developed musculoskeletal symptoms after the second ciprofloxacin dose, and experienced severe Achilles pain within hours of the third dose. Ciprofloxacin was subsequently discontinued, while testosterone therapy was continued unchanged, and the patient's pain and functional impairment gradually improved over several weeks. The continued tolerance of TRT during recovery argues against testosterone itself being the immediate cause of the acute event. The relevance of TRT in this case is instead whether therapeutic androgen exposure could have represented a background susceptibility factor.

Several observational studies have raised the possibility of an association between prescription testosterone and tendon injury. Albright et al. evaluated 423,278 patients with at least three consecutive months of prescribed TRT and an equally sized matched control cohort. The two-year incidence of Achilles tendon injury was 377.8 per 100,000 person-years among patients prescribed TRT compared with 245.8 per 100,000 person-years among controls. After adjustment for relevant comorbidities, TRT exposure was associated with increased odds of Achilles tendon injury, with an adjusted odds ratio of 1.24 [1]. Similar retrospective analyses have reported associations between prescription testosterone and injuries involving the distal biceps, quadriceps, and rotator cuff tendons [2-4]. More recently, androgen therapy among active-duty military personnel was associated with an increased risk of tendon rupture in men, with an adjusted hazard ratio of 1.85, as well as increased rates of Achilles tendon injury [5].

These findings should be interpreted cautiously. Observational database studies demonstrate associations rather than causation and may be affected by differences in physical activity, resistance training, underlying medical conditions, treatment indication, and other unmeasured variables. In addition, prescription records cannot reliably establish adherence, serum testosterone concentrations throughout treatment, or unreported use of additional androgenic compounds. These limitations are particularly important when attempting to extrapolate population-level associations to an individual patient receiving physiologic replacement.

Much of the earlier literature concerning androgens and tendon injury involved supraphysiologic anabolic-androgenic steroid use rather than medically appropriate TRT. Long-term anabolic-androgenic steroid users have demonstrated substantially higher rates of tendon rupture than otherwise similar weightlifters without such exposure [6]. Experimental studies have also suggested that supraphysiologic androgen exposure may alter tendon remodeling and interfere with some structural adaptations to mechanical loading [7]. One proposed explanation is a mismatch between relatively rapid increases in muscle strength and slower adaptation of tendon tissue, although direct effects on collagen organization and extracellular matrix turnover have also been proposed. These findings cannot be assumed to apply to physiologic testosterone replacement.

The relationship between testosterone and tendon tissue is likely more complex than a uniformly harmful effect. Experimental testosterone administration has demonstrated potentially favorable effects on tendon healing in some settings. In a murine rotator cuff repair model, testosterone supplementation improved aspects of histologic healing and functional recovery without compromising biomechanical strength [8]. Taken together, the existing literature suggests that androgen effects on tendon biology may depend on dose, duration, baseline hormonal state, tendon location, and mechanical loading. 

In contrast, fluoroquinolone-associated tendinopathy is a well-established adverse drug effect. Current ciprofloxacin prescribing information in the United States includes tendinitis and tendon rupture within its boxed warning. The Achilles tendon is the most commonly affected site; involvement may be bilateral, and symptoms can occur within hours or days after treatment is initiated [9]. Health Canada has similarly recognized that fluoroquinolone-associated tendinitis and tendinopathy can, in rare cases, be persistent and disabling [10]. European regulatory authorities have restricted systemic fluoroquinolone use because of serious and potentially prolonged adverse effects involving tendons and other musculoskeletal structures [11].

Population-level studies further support this association. Fluoroquinolone exposure has been associated with an increased risk of tendon rupture, with risk influenced by the timing of exposure, cumulative dose, age, and concurrent corticosteroid therapy [12]. Pharmacovigilance analyses have also identified numerous reports of tendon rupture associated with fluoroquinolones, including ciprofloxacin [13]. The rapid onset observed in the present case is therefore notable for its severity but is consistent with the recognized time course of fluoroquinolone-associated tendon toxicity.

Several mechanisms have been proposed for fluoroquinolone effects on tendon tissue. Experimental studies have demonstrated alterations in tenocyte function and extracellular matrix homeostasis, including changes in collagen turnover, matrix metalloproteinase pathways, collagen cross-linking, and other processes involved in maintaining tendon structure [14,15]. These effects provide biological plausibility for an acute medication-related tendon syndrome, although the mechanisms responsible for the substantial variability in individual susceptibility remain incompletely understood.

The present patient lacked several of the commonly recognized factors that can increase the risk of tendon injury. He was in his 30s, had a body mass index within the healthy range, normal renal function, and no history of diabetes mellitus, rheumatologic disease, previous Achilles tendinopathy, or Achilles tendon rupture. He was a nonsmoker and was not receiving systemic corticosteroids or statins. There had also been no recent strenuous physical activity or unusual mechanical loading that would otherwise provide an obvious precipitating explanation. He denied anabolic-androgenic steroid use, use of other androgenic compounds, and aromatase inhibitor therapy. These features do not establish that TRT contributed to the event, but they make consideration of less conventional susceptibility factors reasonable.

Alternative explanations were considered. Mechanical Achilles injury was less likely in the absence of acutely strenuous activity, trauma, or unusual tendon loading. The underlying urinary infection could potentially have contributed to the more generalized musculoskeletal symptoms that initially occurred, including pain involving the knees and other sites. However, the abrupt progression to prominent bilateral Achilles symptoms shortly after ciprofloxacin administration, combined with subsequent improvement following drug withdrawal, was more characteristic of fluoroquinolone-associated tendinopathy. An inflammatory or rheumatologic process was also considered less likely given the patient's lack of relevant history and the close medication-related temporal sequence. There was no clinical evidence of Achilles tendon rupture. Because Achilles tendinopathy is primarily a clinical diagnosis and there were no findings suggesting rupture or another structural complication requiring urgent assessment, imaging was not considered necessary during the acute presentation.

Application of the Naranjo adverse drug reaction probability scale to ciprofloxacin yielded a score of 6, corresponding to a probable adverse drug reaction [16]. Points were supported by the well-established previous reports of fluoroquinolone-associated tendinopathy, onset after ciprofloxacin administration, improvement following drug discontinuation, and the absence of a more compelling alternative explanation for the Achilles syndrome. No rechallenge was performed because re-exposure to ciprofloxacin following a severe suspected tendon reaction would not have been clinically appropriate. The Naranjo assessment supports ciprofloxacin as the likely cause of the acute tendinopathy but does not address whether concurrent TRT modified susceptibility.

The principal hypothesis raised by this case is therefore not that testosterone caused the acute tendon injury, nor that a clinically established drug interaction exists between testosterone and ciprofloxacin. Rather, it is possible that physiologic testosterone exposure could influence baseline tendon characteristics in a manner that modifies vulnerability to a second tendon-toxic exposure. If therapeutic testosterone alters tendon remodeling or adaptation in some individuals, subsequent disruption of extracellular matrix homeostasis by a fluoroquinolone could theoretically produce a clinically significant event at a lower threshold. At present, this remains speculative. The available observational studies evaluate testosterone-associated tendon injury independently of fluoroquinolone exposure, and current regulatory guidance does not identify TRT as a specific risk factor for fluoroquinolone-associated tendinopathy [1-5,9-11].

This report has several limitations. A single case cannot demonstrate that TRT increases the risk of fluoroquinolone-associated tendinopathy or establish an additive or synergistic effect between the two exposures. Diagnostic imaging was not obtained because there was no clinical evidence of tendon rupture, and imaging was not considered necessary for the clinical diagnosis of tendinopathy. Rechallenge with ciprofloxacin was not performed for safety reasons. In addition, the literature associating therapeutic testosterone with tendon injury remains predominantly retrospective and is susceptible to residual confounding. The proposed contribution of TRT should therefore be considered hypothesis-generating rather than causal.

Nevertheless, this case raises a clinically relevant question. Fluoroquinolone prescribing already requires consideration of factors that may increase susceptibility to tendon injury. Emerging observational evidence associating therapeutic testosterone exposure with tendon disorders suggests that concurrent TRT may warrant further investigation as a potential modifier of tendon vulnerability. Current evidence is insufficient to recommend routine avoidance of fluoroquinolones in patients receiving TRT or interruption of testosterone therapy when a fluoroquinolone is clinically necessary. Larger pharmacoepidemiologic studies specifically examining concurrent therapeutic testosterone and fluoroquinolone exposure would be required to determine whether combined exposure is associated with greater tendon risk than fluoroquinolone therapy alone.

## Conclusions

This case describes severe, rapidly developing Achilles tendinopathy following only several doses of ciprofloxacin in a man in his 30s receiving physiologic testosterone replacement therapy. The onset shortly after ciprofloxacin initiation and gradual improvement following ciprofloxacin withdrawal, despite uninterrupted continuation of TRT, support ciprofloxacin as the likely proximate precipitating exposure. Recent observational studies have independently associated therapeutic testosterone exposure with Achilles and other tendon injuries, raising the possibility that TRT could represent an additional susceptibility factor in some patients. However, an additive or synergistic interaction between testosterone and fluoroquinolones has not been established. This hypothesis-generating case highlights the importance of considering the broader tendon-risk profile of patients receiving fluoroquinolones and supports further investigation of concurrent therapeutic androgen and fluoroquinolone exposure.
